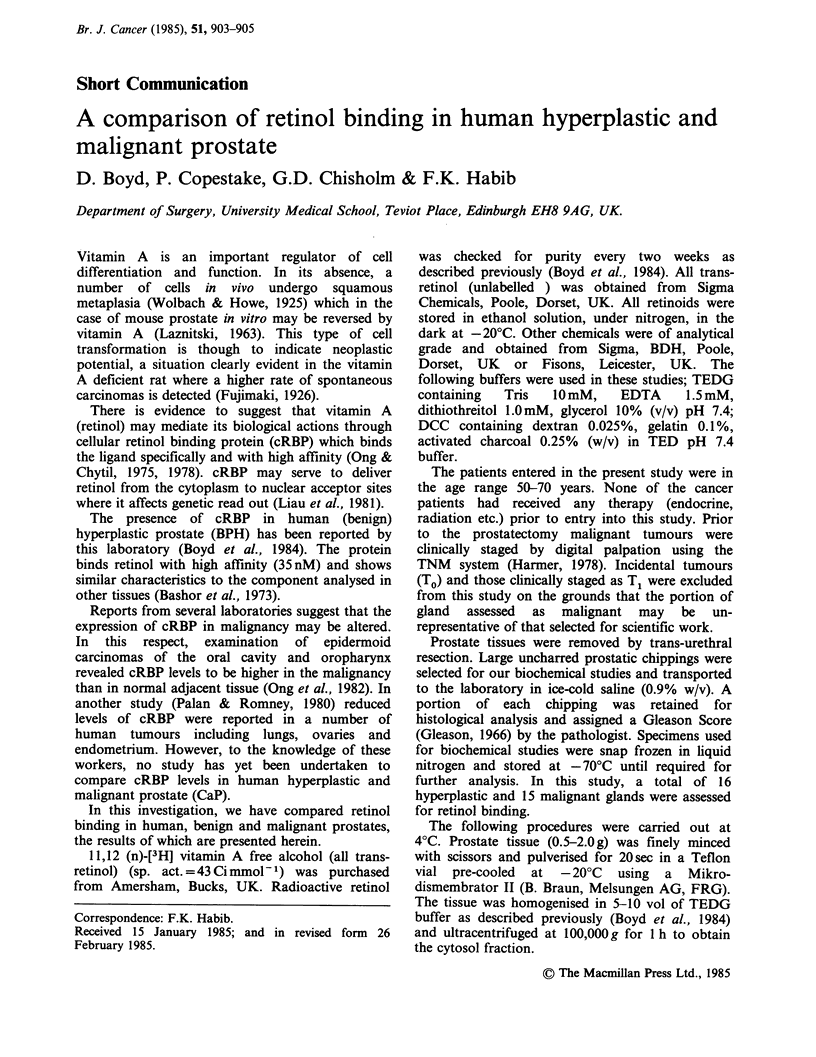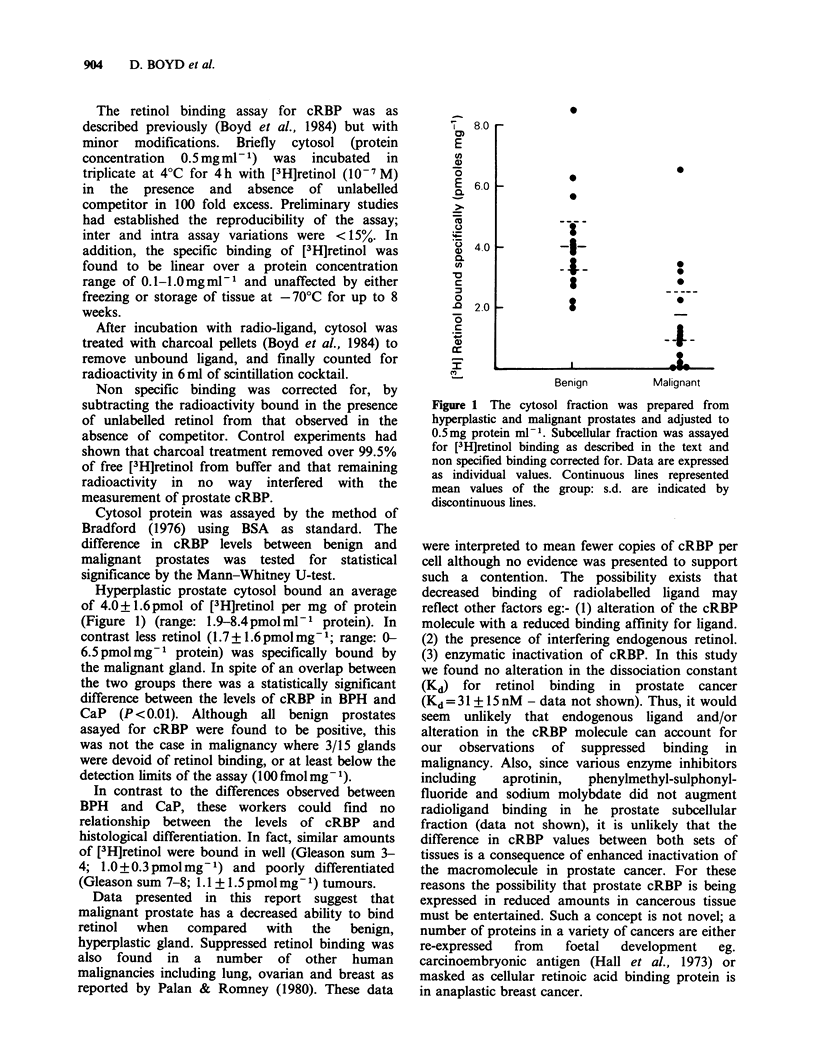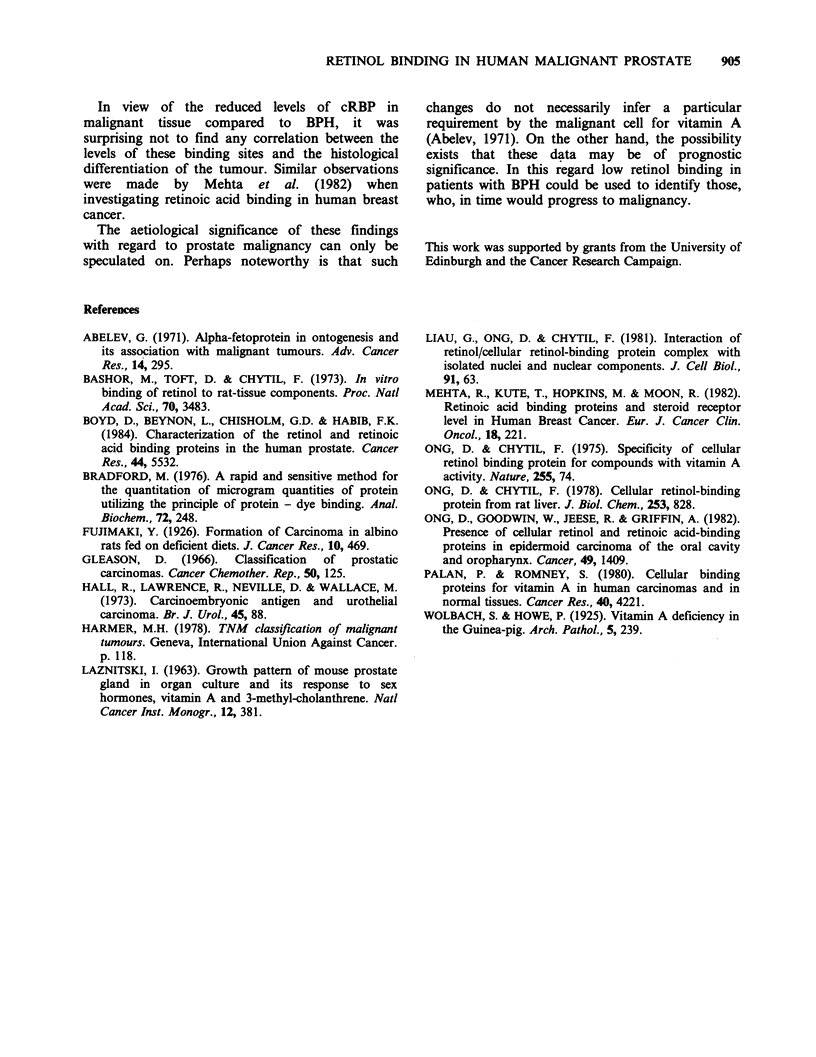# A comparison of retinol binding in human hyperplastic and malignant prostate.

**DOI:** 10.1038/bjc.1985.137

**Published:** 1985-06

**Authors:** D. Boyd, P. Copestake, G. D. Chisholm, F. K. Habib


*Br. J. Cancer* (1985), **51**, 903–905

**Short Communication**

### A comparison of retinol binding in human hyperplastic and malignant prostate

D. Boyd, P. Copestake, G.D. Chisholm & F.K. Habib

*Department of Surgery, University Medical School, Teviot Place, Edinburgh EH8 9AG, UK.*

Vitamin A is an important regulator of cell differentiation and function. In its absence, a number of cells *in vivo* undergo squamous metaplasia (Wolbach & Howe, 1925) which in the case of mouse prostate *in vitro* may be reversed by vitamin A (Laznitski, 1963). This type of cell transformation is though to indicate neoplastic potential, a situation clearly evident in the vitamin A deficient rat where a higher rate of spontaneous carcinomas is detected (Fujimaki, 1926).

There is evidence to suggest that vitamin A (retinol) may mediate its biological actions through cellular retinol binding protein (cRBP) which binds the ligand specifically and with high affinity (Ong & Chytil, 1975, 1978). cRBP may serve to deliver retinol from the cytoplasm to nuclear acceptor sites where it affects genetic read out (Liau *et al.*, 1981).

The presence of cRBP in human (benign) hyperplastic prostate (BPH) has been reported by this laboratory (Boyd *et al.*, 1984). The protein binds retinol with high affinity (35 nM) and shows similar characteristics to the component analysed in other tissues (Bashor *et al.*, 1973).

Reports from several laboratories suggest that the expression of cRBP in malignancy may be altered. In this respect, examination of epidermoid carcinomas of the oral cavity and oropharynx revealed cRBP levels to be higher in the malignancy than in normal adjacent tissue (Ong *et al.*, 1982). In another study (Palan & Romney, 1980) reduced levels of cRBP were reported in a number of human tumours including lungs, ovaries and endometrium. However, to the knowledge of these workers, no study has yet been undertaken to compare cRBP levels in human hyperplastic and malignant prostate (CaP).

In this investigation, we have compared retinol binding in human, benign and malignant prostates, the results of which are presented herein.

11,12 (n)-[$^3$H] vitamin A free alcohol (all trans-retinol) (sp. act. = 43 Ci mmol$^{-1}$) was purchased from Amersham, Bucks, UK. Radioactive retinol was checked for purity every two weeks as described previously (Boyd *et al.*, 1984). All trans-retinol (unlabelled ) was obtained from Sigma Chemicals, Poole, Dorset, UK. All retinoids were stored in ethanol solution, under nitrogen, in the dark at −20°C. Other chemicals were of analytical grade and obtained from Sigma, BDH, Poole, Dorset, UK or Fisons, Leicester, UK. The following buffers were used in these studies; TEDG containing Tris 10 mM, EDTA 1.5 mM, dithiothreitol 1.0 mM, glycerol 10% (v/v) pH 7.4; DCC containing dextran 0.025%, gelatin 0.1%, activated charcoal 0.25% (w/v) in TED pH 7.4 buffer.

The patients entered in the present study were in the age range 50–70 years. None of the cancer patients had received any therapy (endocrine, radiation etc.) prior to entry into this study. Prior to the prostatectomy malignant tumours were clinically staged by digital palpation using the TNM system (Harmer, 1978). Incidental tumours ($T_0$) and those clinically staged as $T_1$ were excluded from this study on the grounds that the portion of gland assessed as malignant may be unrepresentative of that selected for scientific work.

Prostate tissues were removed by trans-urethral resection. Large uncharred prostatic chippings were selected for our biochemical studies and transported to the laboratory in ice-cold saline (0.9% w/v). A portion of each chipping was retained for histological analysis and assigned a Gleason Score (Gleason, 1966) by the pathologist. Specimens used for biochemical studies were snap frozen in liquid nitrogen and stored at −70°C until required for further analysis. In this study, a total of 16 hyperplastic and 15 malignant glands were assessed for retinol binding.

The following procedures were carried out at 4°C. Prostate tissue (0.5–2.0 g) was finely minced with scissors and pulverised for 20 sec in a Teflon vial pre-cooled at −20°C using a Mikro-dismembrator II (B. Braun, Melsungen AG, FRG). The tissue was homogenised in 5–10 vol of TEDG buffer as described previously (Boyd *et al.*, 1984) and ultracentrifuged at 100,000 *g* for 1 h to obtain the cytosol fraction.

Correspondence: F.K. Habib.
Received 15 January 1985; and in revised form 26 February 1985.

© The Macmillan Press Ltd., 1985

The retinol binding assay for cRBP was as described previously (Boyd *et al.*, 1984) but with minor modifications. Briefly cytosol (protein concentration $0.5\,mg\,ml^{-1}$) was incubated in triplicate at 4°C for 4h with [$^3$H]retinol ($10^{-7}$ M) in the presence and absence of unlabelled competitor in 100 fold excess. Preliminary studies had established the reproducibility of the assay; inter and intra assay variations were <15%. In addition, the specific binding of [$^3$H]retinol was found to be linear over a protein concentration range of $0.1–1.0\,mg\,ml^{-1}$ and unaffected by either freezing or storage of tissue at −70°C for up to 8 weeks.

After incubation with radio-ligand, cytosol was treated with charcoal pellets (Boyd *et al.*, 1984) to remove unbound ligand, and finally counted for radioactivity in 6 ml of scintillation cocktail.

Non specific binding was corrected for, by subtracting the radioactivity bound in the presence of unlabelled retinol from that observed in the absence of competitor. Control experiments had shown that charcoal treatment removed over 99.5% of free [$^3$H]retinol from buffer and that remaining radioactivity in no way interfered with the measurement of prostate cRBP.

Cytosol protein was assayed by the method of Bradford (1976) using BSA as standard. The difference in cRBP levels between benign and malignant prostates was tested for statistical significance by the Mann–Whitney U-test.

Hyperplastic prostate cytosol bound an average of $4.0 \pm 1.6$ pmol of [$^3$H]retinol per mg of protein (Figure 1) (range: $1.9–8.4\,pmol\,ml^{-1}$ protein). In contrast less retinol ($1.7 \pm 1.6\,pmol\,mg^{-1}$; range: 0–$6.5\,pmol\,mg^{-1}$ protein) was specifically bound by the malignant gland. In spite of an overlap between the two groups there was a statistically significant difference between the levels of cRBP in BPH and CaP ($P<0.01$). Although all benign prostates asayed for cRBP were found to be positive, this was not the case in malignancy where 3/15 glands were devoid of retinol binding, or at least below the detection limits of the assay ($100\,fmol\,mg^{-1}$).

In contrast to the differences observed between BPH and CaP, these workers could find no relationship between the levels of cRBP and histological differentiation. In fact, similar amounts of [$^3$H]retinol were bound in well (Gleason sum 3–4; $1.0 \pm 0.3\,pmol\,mg^{-1}$) and poorly differentiated (Gleason sum 7–8; $1.1 \pm 1.5\,pmol\,mg^{-1}$) tumours.

Data presented in this report suggest that malignant prostate has a decreased ability to bind retinol when compared with the benign, hyperplastic gland. Suppressed retinol binding was also found in a number of other human malignancies including lung, ovarian and breast as reported by Palan & Romney (1980). These data were interpreted to mean fewer copies of cRBP per cell although no evidence was presented to support such a contention. The possibility exists that decreased binding of radiolabelled ligand may reflect other factors eg:- (1) alteration of the cRBP molecule with a reduced binding affinity for ligand. (2) the presence of interfering endogenous retinol. (3) enzymatic inactivation of cRBP. In this study we found no alteration in the dissociation constant ($K_d$) for retinol binding in prostate cancer ($K_d = 31 \pm 15$ nM – data not shown). Thus, it would seem unlikely that endogenous ligand and/or alteration in the cRBP molecule can account for our observations of suppressed binding in malignancy. Also, since various enzyme inhibitors including aprotinin, phenylmethyl-sulphonyl-fluoride and sodium molybdate did not augment radioligand binding in he prostate subcellular fraction (data not shown), it is unlikely that the difference in cRBP values between both sets of tissues is a consequence of enhanced inactivation of the macromolecule in prostate cancer. For these reasons the possibility that prostate cRBP is being expressed in reduced amounts in cancerous tissue must be entertained. Such a concept is not novel; a number of proteins in a variety of cancers are either re-expressed from foetal development eg. carcinoembryonic antigen (Hall *et al.*, 1973) or masked as cellular retinoic acid binding protein is in anaplastic breast cancer.

**Figure 1** The cytosol fraction was prepared from hyperplastic and malignant prostates and adjusted to 0.5 mg protein $ml^{-1}$. Subcellular fraction was assayed for [$^3$H]retinol binding as described in the text and non specified binding corrected for. Data are expressed as individual values. Continuous lines represented mean values of the group: s.d. are indicated by discontinuous lines.

In view of the reduced levels of cRBP in malignant tissue compared to BPH, it was surprising not to find any correlation between the levels of these binding sites and the histological differentiation of the tumour. Similar observations were made by Mehta *et al.* (1982) when investigating retinoic acid binding in human breast cancer.

The aetiological significance of these findings with regard to prostate malignancy can only be speculated on. Perhaps noteworthy is that such changes do not necessarily infer a particular requirement by the malignant cell for vitamin A (Abelev, 1971). On the other hand, the possibility exists that these data may be of prognostic significance. In this regard low retinol binding in patients with BPH could be used to identify those, who, in time would progress to malignancy.

This work was supported by grants from the University of Edinburgh and the Cancer Research Campaign.

### References

ABELEV, G. (1971). Alpha-fetoprotein in ontogenesis and its association with malignant tumours. *Adv. Cancer Res.*, **14**, 295.

BASHOR, M., TOFT, D. & CHYTIL, F. (1973). *In vitro* binding of retinol to rat-tissue components. *Proc. Natl Acad. Sci.*, **70**, 3483.

BOYD, D., BEYNON, L., CHISHOLM, G.D. & HABIB, F.K. (1984). Characterization of the retinol and retinoic acid binding proteins in the human prostate. *Cancer Res.*, **44**, 5532.

BRADFORD, M. (1976). A rapid and sensitive method for the quantitation of microgram quantities of protein utilizing the principle of protein – dye binding. *Anal. Biochem.*, **72**, 248.

FUJIMAKI, Y. (1926). Formation of Carcinoma in albino rats fed on deficient diets. *J. Cancer Res.*, **10**, 469.

GLEASON, D. (1966). Classification of prostatic carcinomas. *Cancer Chemother. Rep.*, **50**, 125.

HALL, R., LAWRENCE, R., NEVILLE, D. & WALLACE, M. (1973). Carcinoembryonic antigen and urothelial carcinoma. *Br. J. Urol.*, **45**, 88.

HARMER, M.H. (1978). *TNM classification of malignant tumours*. Geneva, International Union Against Cancer. p. 118.

LAZNITSKI, I. (1963). Growth pattern of mouse prostate gland in organ culture and its response to sex hormones, vitamin A and 3-methyl-cholanthrene. *Natl Cancer Inst. Monogr.*, **12**, 381.

LIAU, G., ONG, D. & CHYTIL, F. (1981). Interaction of retinol/cellular retinol-binding protein complex with isolated nuclei and nuclear components. *J. Cell Biol.*, **91**, 63.

MEHTA, R., KUTE, T., HOPKINS, M. & MOON, R. (1982). Retinoic acid binding proteins and steroid receptor level in Human Breast Cancer. *Eur. J. Cancer Clin. Oncol.*, **18**, 221.

ONG, D. & CHYTIL, F. (1975). Specificity of cellular retinol binding protein for compounds with vitamin A activity. *Nature*, **255**, 74.

ONG, D. & CHYTIL, F. (1978). Cellular retinol-binding protein from rat liver. *J. Biol. Chem.*, **253**, 828.

ONG, D., GOODWIN, W., JEESE, R. & GRIFFIN, A. (1982). Presence of cellular retinol and retinoic acid-binding proteins in epidermoid carcinoma of the oral cavity and oropharynx. *Cancer*, **49**, 1409.

PALAN, P. & ROMNEY, S. (1980). Cellular binding proteins for vitamin A in human carcinomas and in normal tissues. *Cancer Res.*, **40**, 4221.

WOLBACH, S. & HOWE, P. (1925). Vitamin A deficiency in the Guinea-pig. *Arch. Pathol.*, **5**, 239.